# Estudio del test de tolerancia a lactosa como alternativa a test de hidrógeno espirado en el estudio de la malabsorción de lactosa

**DOI:** 10.1515/almed-2020-0074

**Published:** 2020-10-30

**Authors:** Teresa Sendino, Amaia Sandúa, Sofía Calleja, Álvaro González, Estibaliz Alegre

**Affiliations:** Servicio de Bioquímica, Clínica Universidad de Navarra, Pamplona, España; Instituto de Investigación Sanitaria de Navarra (IdiSNa), Pamplona, España

**Keywords:** glucosa, hidrógeno, malabsorción lactosa, test de tolerancia

## Abstract

**Objetivos:**

La malabsorción de lactosa se estudia habitualmente mediante el test de hidrógeno espirado (HBT), aunque su realización no es recomendable cuando la concentración de hidrógeno basal (H_2_B) es elevada. Además, la situación actual en relación con el SARS-CoV-2 puede hacer desaconsejable el manejo de muestras de aliento. Objetivo: Evaluar la concordancia del HBT y el test de tolerancia a la lactosa (TTL) en función del H_2_B.

**Métodos:**

Se estudiaron 430 pacientes (40 años, Q1–Q3 = 28–54 años; 66,7% mujeres) con sospecha de malabsorción de lactosa. Se recogieron basalmente y secuencialmente tras la administración de lactosa, muestras de aliento para medir el hidrógeno espirado y de sangre heparinizada para medir la glucemia.

**Resultados:**

El 69,5% de los pacientes tenían H_2_B <10 ppm, el 14,7% valores entre 10 y 20 ppm, y el 15,8% >20 ppm. En los pacientes con H_2_B <20 ppm la concordancia entre el HBT y el TTL era moderada, mejorando siempre al emplear un punto de corte de 15 mg/dL en el TTL. El incremento de hidrógeno y el de glucosa correlacionaron negativamente (r=−0,389; p<0,05). El aumento observado en la glucemia durante el TTL no variaba en función de los niveles de H_2_B registrados en el HBT.

**Conclusiónes:**

El TTL puede ser una alternativa al HBT para evaluar la malabsorción de lactosa cuando los niveles de H_2_B sean elevados o las circunstancias desaconsejen el manejo de muestras de aliento. La mejor concordancia se observa cuando se toma como punto de corte en el TTL de 15 mg/dL.

## Introducción

Los productos lácteos actualmente están presentes en numerosos alimentos y en los adultos representan aproximadamente el 14% de la ingesta calórica en Europa [1]. La lactosa, principal azúcar en la leche, se hidroliza en el intestino delgado por la enzima lactasa-floricina hidrolasa a glucosa y galactosa como paso previo a su absorción. La deficiencia de actividad lactasa intestinal en adultos es bastante frecuente, aunque con importantes variaciones dependiendo de los grupos étnicos, y está asociada a polimorfismos en la región promotora del gen lactasa, principalmente el polimorfismo C/T-13910. Así, los genotipos TT y TC, frecuentes en la población europea, se asocian a la persistencia de la actividad lactasa [2], [3]. Además, la deficiencia de actividad lactasa puede ser secundaria a alteraciones gastrointestinales, como la enfermedad celiaca, gastroenteritis y enfermedad de Crohn [1].

La deficiencia de lactasa causa malabsorción de lactosa en el intestino delgado. La lactosa no absorbida se metaboliza por las bacterias colónicas produciendo gases, como hidrógeno y metano. La sintomatología de esta intolerancia a la lactosa tras la ingesta de leche o derivados incluye dolor abdominal, hinchazón, flatulencia o diarrea [4]. Aproximadamente un tercio de las personas afectadas desarrollan intolerancia a la lactosa [5]. No obstante, la mayoría de individuos con malabsorción de lactosa pueden tolerar ciertas cantidades de lactosa sin manifestar sintomatología debido a la adaptación colónica [6], proceso de adaptación de la microbiota intestinal por el que se promueve la proliferación de bacterias productoras de lactasa cuando se ingieren pequeñas cantidades de lactosa.

Existen diversos métodos para evaluar la malabsorción de lactosa. Aunque el método de referencia consiste en la medida de la actividad lactasa en una biopsia de yeyuno, dada la invasividad de la prueba, los métodos más usados son los indirectos, basados en una sobrecarga con lactosa y la medida a diversos tiempos de la concentración de glucosa en sangre (test de tolerancia de lactosa, TTL) o de hidrógeno espirado (test de hidrógeno en aliento, HBT) [1]. Adicionalmente existen otras pruebas, como el test genético o el test de gaxilosa [1].

El HBT es el método indirecto más utilizado actualmente en el estudio de la malabsorción de lactosa, el de mayor eficacia diagnóstica y el que cuenta con más respaldo bibliográfico [7]. Existen consensos recientes en relación a la metodología empleada y la interpretación de los resultados [8], [9]. Se considera habitualmente como resultado positivo un incremento en el aliento de 20 ppm de H_2_ sobre el valor basal en las tres horas siguientes de la sobrecarga de lactosa [8]. Para esta prueba es necesaria una preparación previa del paciente, que incluye una dieta sin fibra ni lactosa en las 24 horas anteriores a la prueba, y la realización de un enjuague bucal con un colutorio antes de iniciar la prueba. Además, en los días previos, no se deben haber tomado antibióticos ni laxantes, ni tampoco haberse sometido a procedimientos como enemas o colonoscopias que pueden alterar la microbiota intestinal. Habitualmente y bajo cumplimiento del ayuno, el H_2_ basal suele situarse en 7 ± 5 ppm [10]. Una importante limitación de los HBT es su difícil interpretación cuando el H_2_ basal es elevado (>20 ppm), lo que hace que en esas circunstancias se recomiende no continuar la prueba.

Además, recientemente ha aparecido una nueva limitación a la hora de realizar estas pruebas. El virus SARS-CoV-2, causante de la pandemia COVID-19, convierte las muestras de aliento en potencialmente contagiosas al poder contener gotículas con partículas víricas [11], lo que complica la realización de las pruebas en condiciones de seguridad tanto para los pacientes como para el personal sanitario.

Por su parte, el TTL es una prueba más económica, aunque más invasiva [1]. No hay una estandarización metodológica y existen diversos protocolos, así como diferentes puntos de corte para la interpretación de la malabsorción. Aunque generalmente se usa un punto de corte de 20 mg/dL de incremento de concentración de glucosa sobre el basal, algunos autores emplean 25 mg/dL como punto de corte [12] y otros, encuentran una mejor sensibilidad y especificidad usando 15 mg/dL [7], [13]. Una limitación importante es que este test no es válido ni está indicado en pacientes diabéticos [14] y además, un sobrecrecimiento bacteriano puede interferir en su interpretación.

Estudios previos han mostrado que la concordancia entre el HBT y el TTL es moderada [7], [13], [15]. En el presente trabajo pretendemos reevaluar los diferentes puntos de corte empleados en el TTL para una mejor concordancia entre los dos métodos y revisar la utilidad de este test como método alternativo cuando el H_2_ basal sea elevado o cuando las circunstancias hagan no recomendable emplear muestras de aliento, como por ejemplo, para evitar riesgo de contagio con el SARS-CoV-2.

## Materiales y métodos

### Pacientes

Se incluyeron inicialmente en el estudio 516 pacientes a los que se había realizado un estudio de malabsorción de lactosa dentro de su manejo asistencial que incluía tanto un HBT como un TTL, durante del periodo de 3 años (Marzo 2007–Marzo 2010). Para evitar desviaciones por posibles problemas técnicos, fueron excluidas aquellas pruebas en las que el H_2_ fue indetectable en todas las muestras de aliento. También se excluyeron aquellas pruebas con glucemias basales superiores a 126 mg/dL por la posible alteración en el metabolismo de la glucosa que puede interferir en la interpretación. El número final de pacientes incluidos fue de 430 (mediana edad=40 años, Q1–Q3 = 28–54 años; 66,7% mujeres). Los pacientes habían llevado una dieta pautada sin fibra ni lactosa durante las 24 h anteriores a la prueba, sin consumo de tabaco el día que se realizó la misma y acudieron en ayunas. Tampoco se permitió la administración de antibióticos, laxantes o enemas así como la realización de colonoscopias en los 7 días previos a la prueba. Previamente al inicio de la prueba los pacientes realizaron un enjuague bucal con colutorio. El estudio ha recibido la correspondiente aprobación del Comité de Ética Institucional.

### Test de sobrecarga de lactosa

Como estímulo se administraron 50 g de lactosa por vía oral disueltos en 200 mL de agua. Esta dosis no se corresponde con la recomendada actualmente para el HBT, pero en su momento era una dosis empleada habitualmente en la realización del TTL. Para el HBT se recogieron muestras de aire espirado, basal y cada 30 minutos durante 3 horas. La concentración de H_2_ se midió en un equipo Breath Tracker H2+ (QuinTron, Milwaukee, Estados Unidos). Una elevación de la concentración de hidrógeno superior a 20 ppm respecto al valor basal se consideró indicativa de malabsorción de lactosa [8]. Para el TTL se obtuvieron muestras de sangre periférica heparinizada, basal y secuencialmente cada 30 minutos durante 2 horas. Tras centrifugación se determinaron los niveles plasmáticos de glucosa con el método glucosa-oxidasa en un equipo modular P (Roche Diagnostics, Mannheim, Alemania). Se calculó el incremento máximo de la glucemia durante la prueba respecto a la concentración inicial. Se analizaron tres posibles puntos de corte para malabsorción según el incremento respecto al basal: 25 mg/dL, 20 mg/dL y 15 mg/dL.

### Análisis estadístico

Los datos están expresados como mediana y rango intercuartílico. La distribución no-gaussiana de los datos se comprobó con el test de normalidad de Kolmogorov-Smirnov. La comparación de datos entre grupos se realizó mediante el test de Kruskal–Wallis, seguido del test de comparaciones múltiples de Dunn. Las correlaciones se evaluaron mediante el coeficiente de correlación de Spearman. La comparación de frecuencias entre grupos se realizó mediante la prueba chi cuadrado. El nivel de concordancia se evaluó con el índice kappa de Cohen considerando un valor de 0,81–1,00 “muy bien”; 0,61–0,80 “bien”; 0,41–0,60 “moderado” y 0,21–0,40 “bajo” [16]. Los datos estadísticos se analizaron usando IBM SPSS Statisics v20. Un valor *P* bilateral de <0,05 se consideró estadísticamente significativo.

## Resultados

De los 516 pacientes iniciales se seleccionaron 430, excluyéndose aquellos que tenían una glucemia basal superior a 126 mg/dL y aquellos en los que no se detectaba producción de H_2_ en el aliento en ningún momento de la prueba. Los resultados del HBT y TTL se exponen en la Tabla 1. De manera global con el HBT se observó una malabsorción de lactosa en el 26,7% de los pacientes, un 57,4% con el TTL usando un punto de corte de 25 mg/dL, un 43,5% usando un punto de corte de 20 mg/dL, y un 32,3% si el punto de corte elegido era de 15 mg/dL.

**Tabla 1:** Relación entre el test de hidrógeno espirado (HBT) y el test de tolerancia a lactosa (TTL) usando diferentes puntos de corte de incremento de la glucemia respecto al H_2_ basal para indicar malabsorción. Se considera malabsorción en el HBT un incremento de H_2_ respecto al basal mayor de 20 ppm.

H_2_ basal menor de 10 ppm						
			HBT		Total	Índice kappa (s95% IC)
			Normal	Malabsorción		
TTL	25 mg/dL	Normal	127	12	139 (46,5%)	0,36 (0,27–0,45)
		Malabsorción	87	73	160 (53,5%)	
	20 mg/dL	Normal	159	19	178 (59,6%)	0,46 (0,36–0,56)
		Malabsorción	55	66	121 (40,4%)	
	15 mg/dL	Normal	180	24	204 (68,2%)	0,54 (0,44–0,64)
		Malabsorción	34	61	95 (31,8%)	
Total			214 (71,2%)	85 (28,8%)	n=299	

H_2_ basal entre 10 y 20 ppm						
			HBT		Total	Índice kappa (95% IC)
			Normal	Malabsorción		
TTL	25 mg/dL	Normal	23	1	24 (38,1%)	0,24 (0,09–0,4)
		Malabsorción	26	13	39 (61,9%)	
	20 mg/dL	Normal	32	2	34 (54,0%)	0,37 (0,16–0,58)
		Malabsorción	17	12	29 (46,0%)	
	15 mg/dL	Normal	40	3	43 (68,3%)	0,52 (0,29–0,75)
		Malabsorción	9	11	20 (31,7%)	
Total			49 (77,8%)	14 (22,2%)	n=63	

H2 basal mayor de 20 ppm			
			Total
TTL	25 mg/dL	Normal	20 (29,4%)
		Malabsorción	48 (70,6%)
	20 mg/dL	Normal	31 (45,6%)
		Malabsorción	37 (54,4%)
	15 mg/dL	Normal	44 (64,7%)
		Malabsorción	24 (35,3%)
Total			n=68

Considerando la concentración de H_2_ basal, se observó un grupo de 299 (69,5%) pacientes con niveles basales de hidrógeno menor de 10 ppm, un segundo grupo de 63 (14,7%) pacientes que tenía niveles basales entre 10 y 20 ppm y un tercer grupo de 68 (15,8%) pacientes que tenía unos niveles basales de hidrógeno superiores a 20 ppm.

**Figura 1:**
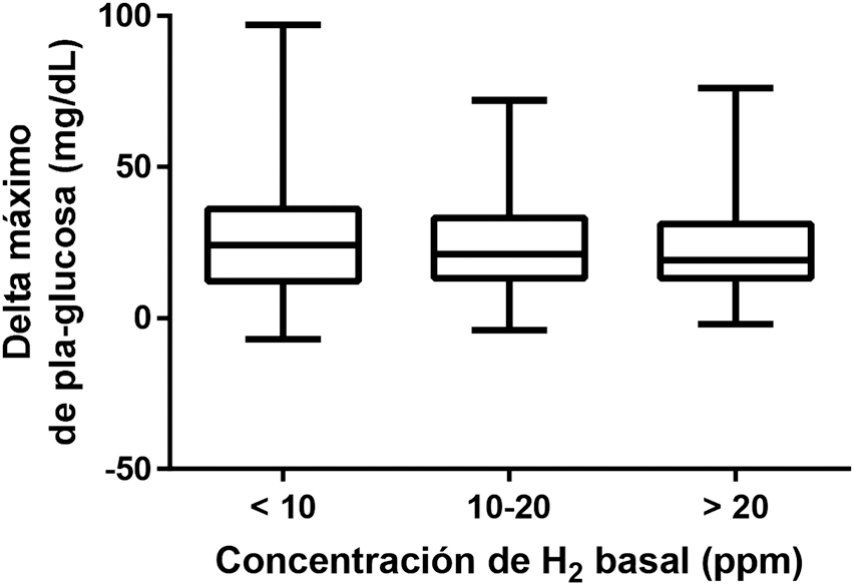
Delta máximo observado en la glucemia plasmática (mg/dL) durante el test de tolerancia a lactosa en función de los niveles basales de H_2_.

De los 362 pacientes con una concentración de H_2_ basal inferior a 20 ppm, un 27,3% presentaba malabsorción según el HBT. Mediante el TTL se observó malabsorción en el 55% de los casos cuando el punto de corte era 25 mg/dL, en el 41,4% usando como punto de corte 20 mg/dL, y en el 31,7% cuando el punto de corte usado era de 15 mg/dL. El análisis de concordancia entre el HBT y el TTL tenía un índice kappa de 0,33 (95% IC: 0,25–0,41) usando un punto de corte de 25 mg/dL de glucosa, 0,44 (95% IC: 0,35–0,53) usando un punto de corte de 20 mg/dL y 0,51 (95% CI: 0,42–0,58) con un punto de corte de 15 mg/dL. Se observó una correlación negativa entre el delta máximo de H_2_ y el de glucosa, de manera que a mayor aumento de H_2_, menor era la elevación de la glucemia (r=−0,389; p<0,05).

En el subgrupo de 299 pacientes con H_2_ basal menor de 10 ppm, el 28,8% presentaban malabsorción según el HBT. En el caso del TTL, el porcentaje se situaba en un 53,6% con un punto de corte de 25 mg/dL, en un 40,4% usando un punto de corte de 20 mg/dL, y en un 31,8% si el punto de corte usado era de 15 mg/dL. La concordancia entre el HBT y el TTL presentaba un índice kappa de 0,36 (95% IC: 0,27–0,45) con un punto de corte de 25 mg/dL, de 0,46 (95% IC: 0,36–0,56) usando un punto de corte de 20 mg/dL y 0,54 (95% CI: 0,44–0,64) usando un punto de corte de 15 mg/dL.

En el subgrupo de 63 pacientes con concentraciones de H_2_ basal entre 10 y 20 ppm, el 22,2% de ellos mostraban posteriormente incrementos superiores a 20 ppm durante el HBT, indicando malabsorción. Analizando los resultados del TTL un 61,9% mostraban malabsorción empleando un punto de corte de 25 mg/dL, un 46,0% usando un punto de corte de 20 mg/dL, mientras que el porcentaje era de un 31,7% usando un punto de corte de 15 mg/dL. El análisis de concordancia entre el HBT y el TTL mostraba un índice kappa de 0,24 (95% IC: 0,09–0,4) si se empleaba como punto de corte 25 mg/dL, un 0,37 (95% IC: 0,16–0,58) usando un punto de corte de 20 mg/dL y de 0,52 (95% CI: 0,29–0,75) usando un punto de corte de 15 mg/dL.

Por último, en el grupo de 68 pacientes con H_2_ basal superior a 20 ppm, un 70,6% manifestaba malabsorción según el TTL con un punto de corte de 25 mg/dL, un 54,4% usando un punto de corte de 20 mg/dL y un 35,3% si se usaba un punto de corte de 15 mg/dL.

Aunque el porcentaje de sujetos con malabsorción según el TTL, independientemente del punto de corte empleado, era mayor en los pacientes con H_2_ basal superior a 20 ppm, respecto a los pacientes con niveles basales menores de 10 ppm o entre 10 y 20 ppm, esta diferencia solo fue significativa en el caso de emplear 25 mg/dL (p<0,01) y 20 mg/dL (p<0,05) como punto de corte.

Analizando cuantitativamente el aumento de la glucemia alcanzado durante el TTL se observó que el incremento máximo no variaba significativamente en función de los valores de H_2_ basal (menor 10 ppm, entre 10 y 20 ppm, y mayor de 20 ppm) (See Figura 1).

## Discusión

Este es uno de los estudios para evaluar la concordancia entre los métodos indirectos de análisis de malabsorción de lactosa, HBT y TTL, con mayor número de pacientes incluidos, [7], [15], [17]. Hemos comprobado que, en conjunto, existe una concordancia moderada entre los dos test indirectos analizados. Esta diferencia puede ser debida a que mientras el HBT depende de la flora fermentativa del intestino grueso [18], el TTL se ve influido por la respuesta fisiológica a la glucosa. No obstante, hemos observado que la mejor concordancia entre ambas pruebas se alcanza usando un punto de corte de 15 mg/dL para el TTL que, por otra parte, ha demostrado tener una mayor eficacia diagnóstica respecto al punto de corte de 20 mg/dL [13].

El HBT es el método más ampliamente utilizado y tiene mayor eficacia diagnóstica, con una sensibilidad media del 77,5% y una especificidad del 97,6% [7], [9]. No obstante, pueden existir falsos negativos por la incapacidad de producir H_2_ o la toma de antibióticos, y falsos positivos por el sobrecrecimiento bacteriano [1]. Una limitación de los HBT es que no se recomienda su uso cuando la concentración de H_2_ basal es elevada [1], e incluso algunos autores ponen el límite en un H_2_ basal entre 10–16 ppm [10]. No hemos observado cambios en el grado de elevación de la glucemia en función de la concentración de H_2_ basal. Ello puede ser debido a que no se ve afectada por algunos factores que causan esta elevada producción basal de H_2_, como puede ser no seguir la dieta previa o la liberación de H_2_ atrapado previamente en el intestino [19]. Debido a que el comportamiento del TTL es independiente de los niveles de H_2_ basal, el TTL puede ser una alternativa al HBT en estos pacientes con elevado H_2_ basal en los que no está recomendado su realización o interpretación.

El TTL también podría ser útil en aquellos pacientes no productores de H_2_,que son una pequeña proporción de la población [20]. No obstante, para identificar a estos individuos existe la posibilidad de medir simultáneamente los niveles de metano en el aire espirado, aunque esta opción requiere de equipos más sofisticados y su medida no está tan estandarizada en relación a la malabsorción.

Además, la coyuntura actual ha hecho que las muestras de aliento hayan adquirido un carácter de mayor peligrosidad, ya que los aerosoles de pacientes con la COVID-19 son importantes vehículos de transmisión del virus SARS-CoV-2 al poder contener una elevada carga viral [21]. Por tanto, el procedimiento de obtención y el manejo de las muestras en los HBT y su procesamiento manual, pueden considerarse de riesgo y, por tanto, es importante proceder con las adecuadas medidas de contención de la enfermedad en el laboratorio [22], para proteger tanto a los profesionales sanitarios como al resto de pacientes. Sin embargo, el virus se detecta en la sangre en un bajo porcentaje de pacientes con la COVID-19 y los procedimientos de manejo de muestras y análisis de glucosa suelen estar muy automatizados, con lo que el riesgo de contagio es mucho menor. Por tanto, este tipo de muestras es mucho más adecuado para evitar el riesgo de contagio.

En este estudio la cantidad de lactosa en el estímulo es mayor que la recomendada para el HBT [8], pero era una cantidad habitualmente usada en el TTL [1]. El uso de 50 g de lactosa (aproximadamente la contenida en un litro de leche) es superior a la ingerida habitualmente, y puede causar mayor sintomatología, especialmente dolor abdominal y diarrea. Al contrario de lo que ocurre para el HBT, no existe un consenso en el protocolo de realización del TTL, en cuanto a la dosis de lactosa, tipo de espécimen, intervalo de recogida de muestra o el punto de corte empleado. Así, se ha propuesto acortar la duración de la prueba a una hora [23] y realizar el análisis de la glucemia en sangre capilar respecto a la sangre venosa encontrándose una concordancia modesta [24]. Recientemente se ha desarrollado un test estudio de malabsorción de lactosa con 4-Galactosilxilosa, aunque existen pocos estudios comparativos [25]. Por otra parte, el test genético analizando el polimorfismo C/T-13910 tiene el inconveniente de que es más caro, y solamente identifica a los individuos con una causa primaria de descenso de expresión del gen, pero no una hipolactasia por otras causas [17]. Además, no se tiene información clínica del paciente tras la exposición a la lactosa.

En conclusión, en este estudio hemos observado que el TTL puede ser una alternativa al HBT cuando la concentración basal de H_2_ es elevada y no sea adecuado usar el HBT para evaluar la malabsorción de lactosa, siendo el punto de corte de 15 mg/dL el más adecuado para unos resultados comparables. No obstante, hay que tener en cuenta que con la reducción del punto de corte a 15 mg/dL se diagnosticará la malabsorción de lactosa en un menor número de pacientes.
